# Unusual case of ruptured sinus of valsalva aneurysm in a pregnant woman

**DOI:** 10.11604/pamj.2017.27.271.9741

**Published:** 2017-08-10

**Authors:** Salma Charfeddine, Dorra Abid, Faten Triki, Leila Abid, Samir Kammoun, Imed Frikha

**Affiliations:** 1 Department of Cardiology, Hedi Chaker University Hospital, Sfax, Tunisia; 2 Faculty of Medicine, Sfax, Tunisia; 3 Department of Cardiovascular and Thoracic Surgery, Habib Bourguiba University Hospital, Sfax, Tunisia

**Keywords:** Sinus of valsalva aneurysm, ruptured aneurysm, transthoracic echocardiography diagnosis, patch repair

## Abstract

Sinus of Valsalva aneurysms are extremely rare, and usually of a congenital nature. There are few documented cases of this condition during pregnancy, which renders unclear the therapeutic options. We here report the case of a 26 years old pregnant woman who was referred to our cardiac center for the evaluation of a heart murmur. The two-dimensional transthoracic echocardiography allowed quickly to establishthe diagnosis of a ruptured sinus of Valsalva aneurysm into the right ventricle. A successful surgical correction of the ruptured aneurysm was performed with patch repair.

## Introduction

Sinus of Valsalva (SV) aneurysm is a rare cardiac anomaly [1]. The SV aneurysms usually remain asymptomatic unless they are complicated by rupture. The rupture may be spontaneous, after trauma, extreme physical exercise or due to endocarditis [1-3]. We report an unusual case of the right sinus of Valsalva aneurysm rupture into the right ventricle in a pregnant woman.

## Patient and observation

**Figure 1:**
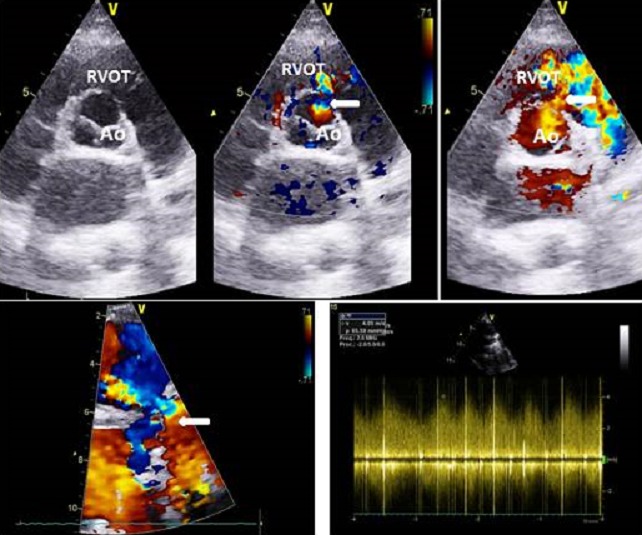
2D-TTE and Doppler interrogation show rupture of the wall of the right coronary sinus of valsalva and flow of blood into the right ventricle

A 26 year-old pregnant woman was referred to our institution for cardiac evaluation due to the presence of a continuous heart murmur. She was 26 weeks pregnant. She had an uncomplicated previous pregnancy. A history of a cardiac murmur first detected during her childhood was reported. She had undergone two-dimensional trans-thoracic echocardiography (2D-TTE) at that time and knew that she had a restrictive ventricular septal defect (VSD). She remained asymptomatic for almost 12 years. Recently, the patient reported the presence of a cardiac thrill and the onset of chest pain after an extreme physical effort to carry a heavy load of 25 kilograms. The precordium palpation revealed a systolo-diastolic thrill. The blood pressure was 125/50 mmHg. The heart auscultation revealed a continuous murmur along the left sternal border. A 2D-TTE showed a large communication between the right SV and the right ventricle outflow tract (RVOT) with left to right shunt (Figure 1).

**Figure 2:**
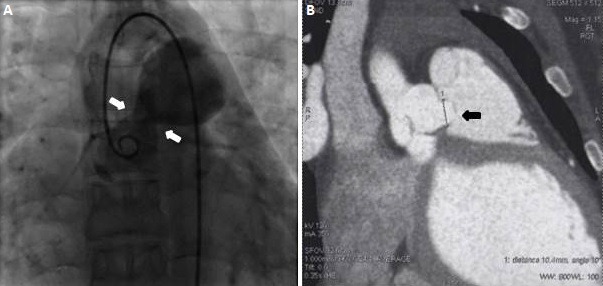
Angiograms (aortography and CT angiography) show ruptured right sinus of Valsalva aneurysm into the right ventricle

**Figure 3:**
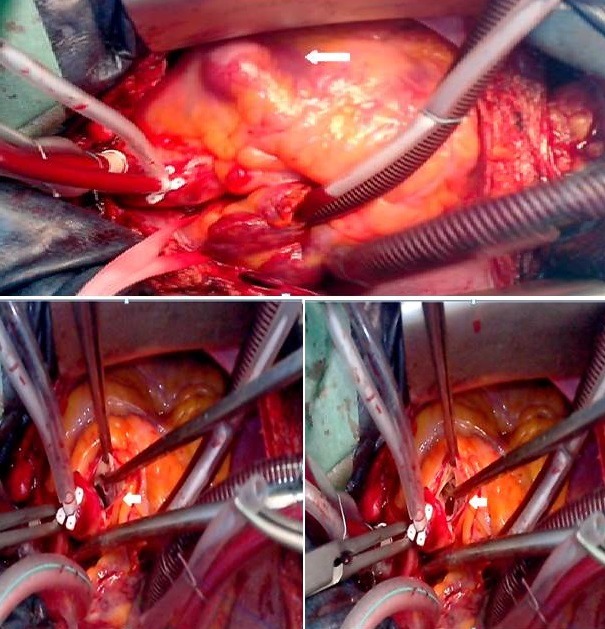
Surgical views of the ruptured sinus of Valsalva aneurysm into the right ventricle and the interventricular septal defect

**Figure 4:**
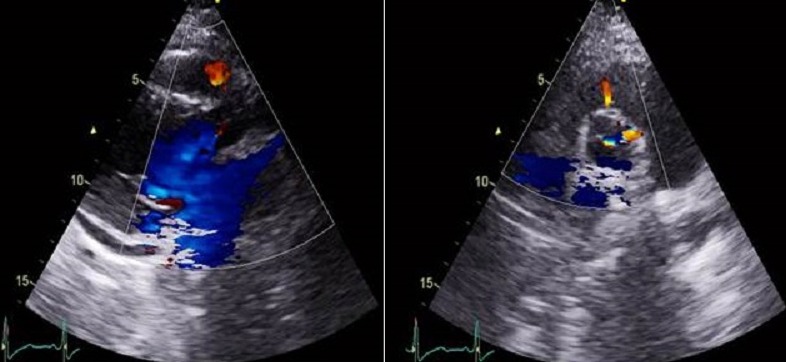
Post operative 2D-TTE shows trivial residual communication between the aorta and the right ventricle

The right cardiac chambers were not dilated. The left ventricular ejection fraction was normal. The aortic valve was tricuspid with mild aortic regurgitation. Diameters of the aortic root and ascending aorta were within normal limits. The pulmonary valve was normal and there was evidence of pulmonary hypertension, with an estimated pulmonary artery systolic pressure of 45 mmHg. The end-diastolic reverse flow velocity in the descending aorta just beneath the aortic isthmus was 30 cm/sec. The patient was monitored closely with regular follow-up at our cardiology institution throughout the remainder of her pregnancy and had an uncomplicated scheduled caesarean delivery. A subsequent echocardiogram after delivery showed similar findings. The configuration of the aneurysm was consistent with the contrast filled sinus demonstrated by aortography, with a normal coronary sinus and a mobile aneurysm protruding into the right ventricle (Figure 2a). The chest computed tomography (CT) scan demonstrated also the presence of a ruptured right SV aneurysm into the right ventricle (Figure 2b). The surgical repair was advised. At surgery, a ruptured right coronary SV aneurysm into the right ventricle with a sub-aortic VSD were visualized. The VSD was not diagnosed at the preoperative time. The operative procedure included the closure of the aorto-ventricular communication and the VSD with patches (Figure 3). The patient had an uneventful postoperative course. At 1-month and at one year follow-up, the patient was asymptomatic. A 2D-TTE showed a normal left ventricular ejection fraction and trivial residual communication between the aorta and the right ventricle (Figure 4).

## Discussion

Sinus of Valsalva aneurysm is a very uncommon cardiac anomaly that can be either congenital or acquired. Its incidence ranges from 0.1% to 3.5% of all congenital heart defects [1]. The morphology of congenital SV aneurysm is attributed to the absence of muscular and elastic tissue in the aortic wall of the SV [3]. The association between the right SV aneurysms and VSD, as reported in our case, is probably not accidental. It has been proposed that the defect results from incomplete fusion between the right and left distal bulbous septum during the foetal development [3]. Aneurysms may arise from the right coronary sinus (65%-85%), the noncoronary sinus (10%–30%) and rarely, the left coronary sinus (1%-5%) [2]. The rupture of the SV aneurysm most often occurs into the right ventricle followed by right atrium and rarely into the other cardiac chambers [2]. The aneurysms rupture occurs in previously healthy young adults and causes acute symptoms in only one-third of patients [4].

This reported case was presented with a complex management situation. There are few previous cases reported in the literature regarding this abnormality in a pregnant patient [5-7]. Our patient had a previous successful pregnancy in the context of a left-to-right shunt, given her reported history of a congenital cardiac defect since childhood. Therefore, it is not clear when the aneurysm has ruptured. The rupture was probably precipitated by the intensive physical effort made during pregnancy. In fact, the physiologic changes that occur with pregnancy increase the risk in patients with an anatomic cardiac defect [6]. Since the patient was already haemodynamically affected, caesarean section was the preferred method of delivery. One case has been reported previously in which a rupture of the SV occurred at 37 weeks’ gestation and was successfully repaired one week postpartum [7]. It is not clear when surgery should be performed in an asymptomatic patient with a ruptured SV aneurysm. The mortality rate is likely high in patients after rupture when surgical correction is not achieved. In our case, the patient was asymptomatic, surgery was not undertaken during pregnancy, and she was closely monitored without any complications. Few days after delivery, she was successfully operated.

Before the introduction of echocardiography, the diagnosis of a ruptured SV aneurysm in the living patient was rare, with most of the reports coming from autopsy or surgery. Nowadays, the TTE frequently establish the diagnosis and provide very detailed information to the surgeon, as was done in our case [8]. In our report, there was no associated significant aortic valvular regurgitation. But, the end-diastolic reverse flow velocity in the descending aorta was > 20 cm/sec. Thereby, the rupture of the right SV aneurysm into cardiac chambers with important left to right shunt should always be remembered by the clinician as an unusual etiology of a common aortic valvular lesion like severe acute aortic regurgitation. Since the ruptured SV aneurysm into cardiac chambers during pregnancy was unusual, CT scan and aortography were also made in this case to confirm the diagnosis and to detect potential coexisting lesions. We thought that a multimodality imaging could be indicated especially if there was a persisting doubt. In fact, the cardiac angiography, CT scan and magnetic resonance imaging (MRI) might be useful in diagnosing the coexisting cardiac lesions more precisely and evaluating coronary perfusion prior to surgery [9]. The trans-esophageal echocardiography (TEE) could also be very helpful for diagnosing ruptured and in tact aneurysm and could provide a great guidance for intra operative repair. It provided information regarding involved sinuses, protrusion, and associated shunt or coexisting cardiac abnormalities [9]. Considering cost efficiency and radiation, we suggest in similar cases the use of 2D- TTE, eventually TEE and 3D- reconstructions, if available. More exams, such as cardiac angiography, CT scan and MRI should not be indicated in all cases.

The surgical treatment of ruptured aneurysm remains the gold standard. Several authors recommended using patch to close SV aneurysm in all cases because it avoids deforming the aortic valve and reduces stress on the suture line [10]. Although surgery is still the treatment of choice, transcatheter closure techniques have currently added to the spectrum of nonsurgical alternatives for repair. Since 1994, multiple reports have described different approaches for percutaneous closure of SV aneuvrysm using septal occluder device, ductal occluder and Amplatzer vascular plug [9]. Albeit not without complications, transcatheter closure techniques are growing in popularity over open surgery.

## Conclusion

The rupture of the right sinus of Valsalva aneurysm into cardiac chambers in a pregnant woman is unusual and presents a complex management situation. The diagnosis can be made precisely by TTE and TEE. Cardiac angiography, CT scan and MRI can be used as supplemental or confirmatory tests. Although the transcatheter closure techniques are increasingly used, the surgical repair remains the treatment of choice and is associated with a low operative risk.

## Competing interests

The authors declare no competing interests.
